# 肺腺癌驱动基因研究相关进展

**DOI:** 10.3779/j.issn.1009-3419.2013.02.06

**Published:** 2013-02-20

**Authors:** 宗德 张

**Affiliations:** 1 101149 北京，首都医科大学附属北京胸科医院，北京市结核病胸部肿瘤研究所，肿瘤内科 Department of Medical Oncology, Beijing Chest Hospital, Capital Medical University, Beijing Tuberculosis and Thoracic Tumor Research Institute, Beijing 101149, China; 2 101149 北京，首都医科大学附属北京胸科医院，北京市结核病胸部肿瘤研究所，分子生物学实验室 Molecular Biological Laboratory, Beijing Chest Hospital, Capital Medical University, Beijing Tuberculosis and Thoracic Tumor Research Institute, Beijing 101149, China

**Keywords:** 肺肿瘤, 腺癌, 癌基因, Lung neoplasms, Adenocarcinoma, Oncogenes

## Abstract

近来研究证实肺腺癌具有与致癌作用及靶向药物疗效有关的独特分子特征，这些分子改变被视为驱动基因，负责恶性病变的发生和维持。目前发现约50%肺腺癌携带驱动基因，其中EGFR通路起重要作用。本文将对肺腺癌驱动基因的意义及相关研究进行综述。

## Abstract (en)

The adenocarcinoma of the lung has recently shown peculiar molecular characteristics, which relate with both carcinogenesis and response to targeted drugs. Several molecular alterations have been defined as "driver oncogenes" responsible for both the initiation and maintenance of the malignancy. So far, 50% of lung adenocarcinoma has been found harbouring driver oncogenes, in which epidermal growth factor receptor pathway plays important roles. In this review, we will report mainly the advances on significance of some latest driver mutations.

全球范围内，肺癌居癌症死亡原因的首位。肺癌包括小细胞肺癌和非小细胞肺癌（non-small cell lung cancer, NSCLC），NSCLC占80%，分为不同的组织学类型，包括腺癌、鳞癌、大细胞癌、腺鳞癌、肉瘤样癌等，其中腺癌占50%以上。肺癌患者远期生存率低，5年生存率仅为10%-15%，当今急需更有效的治疗策略。

“Oncogene addiction”是指肿瘤细胞的发生、生存和增殖依赖于活化的癌基因的现象，这些异常的分子信号也称为驱动癌基因（driver oncogenes）。癌细胞需要驱动癌基因持续发挥功能，而正常细胞则不需要，因此，癌基因可作为明确的治疗靶点，使靶向药物特异性地杀伤肿瘤细胞，而不损伤正常细胞。过去的十几年里，许多研究在发现驱动基因方面取得了明显进步，以驱动基因为靶点的药物治疗也获得了较为明显的疗效，多项研究^[1, 2]^证实表皮生长因子受体酪氨酸激酶活性抑制剂（epidermal growth factor receptor tyrosine kinase inhibitor, EGFR-TKI）治疗*EGFR*基因突变患者的疗效明显优于传统的化疗和野生型患者。2007年，日本学者在NSCLC中发现的棘皮动物微管相关蛋白样4-间变淋巴瘤激酶融合基因（echinodern microtubule-associated protein-like 4 anaplastic lymphoma kinase, *EML4*-*ALK*）是一个新的驱动基因，以其为靶点的药物克唑替尼（crizotinib）在ALK阳性的晚期NSCLC患者中获得了很好的疗效。本文将对肺腺癌驱动基因的研究进展作一综述。

## 1 肺腺癌各驱动基因的意义

### 1.1 EGFR

EGFR是一个170 kDa的酪氨酸跨膜受体，跨膜酪氨酸激酶ErbB家族4个成员之一，也称为HER1或ErbB1，该家族还包括HER2、HER3和HER4。同源配体与胞外受体结合，导致两个EGFR同型二聚体或异型二聚体结合，致EGFR胞内部分自动磷酸化，激活酪氨酸激酶。随后，细胞内的多个通路包括RAS/RAF/MEK/MAPK、PI3K/AKT/mTOR相继激活，共同促进癌细胞增殖、生长、侵袭、转移及肿瘤血管生成。2004年有研究首次报告EGFR受体酪氨酸激酶区域突变。NSCLC中EGFR最常见的突变有两个，一个是外显子19缺失（LREA缺失，delE746-A750），占45%；另一个是外显子21上的突变，即858密码子亮氨酸替代精氨酸（L858R），占40%。约10%高加索人和近50%亚裔患者存在*EGFR*敏感突变^[3]^，在肺腺癌、不吸烟、女性、亚裔中频率更高^[4]^。T790M（苏氨酸-790-蛋氨酸）突变与TKI治疗的耐药有关，研究^[5]^发现50%疾病进展患者有此种突变，它可能是继发性突变，也可能是治疗前在小的克隆中已经存在的突变（此种约占5%）。另外还存在几种少见的敏感突变和耐药突变，敏感突变有外显子18的G719C、G719S、G719A突变（5%），外显子20的V756A、T783A突变（＜1%），耐药突变有外显子19 D761Y突变（＜1%）^[6]^。2012年NCCN指南推荐EGFR-TKI用于有*EGFR*敏感突变的非鳞NSCLC患者的一线治疗，无突变或突变状况不明的非鳞患者的维持治疗，非选择的PS评分为0分-2分的非鳞患者的二线及三线治疗及有*EGFR*敏感突变的PS评分为3分-4分患者的二线和三线治疗。

荧光原位杂交（fluorescence in situ hybridization, FISH）方法发现30%-60%的晚期NSCLC的*EGFR*基因拷贝数增加，免疫组化方法发现晚期NSCLC中EGFR蛋白表达50%-90%，目前EGFR拷贝数及蛋白表达的预测TKI疗效的作用还存在争议，*EGFR*基因突变仍是最强的预测疗效的因素^[7]^。

EGFR通路主要包括两个下游信号通路：RAS/RAF/MEK和PI3K/AKT/mTOR。

### 1.2 RAS/RAF/MEK通路

#### 1.2.1 KRAS（kirsten-rous avian sarcoma）

RAS家族蛋白有3个编码基因：*HRAS*、*KRAS*和*NRAS*，这3种基因在人类肿瘤上均很常见。研究^[8, 9]^显示，北美腺癌患者中*KRAS*突变频率约为25%，亚裔患者*KRAS*突变率约5%。腺癌突变率明显高于其它组织类型，在吸烟患者多见^[10]^。*KRAS*突变位点为密码子12、13和61，90%突变发生在外显子2的12密码子^[11]^。多项研究证实，*KRAS*突变是肺癌患者独立的预后因素，突变患者的预后较KRAS野生型差^[12]^，突变者对TKI耐药，但并不影响化疗的疗效^[8]^。目前尚无针对KRAS的靶向治疗药物，靶向药物联合化疗或联合抑制PI3K和MEK的靶向药物治疗*KRAS*突变患者正处于临床研究中。

#### 1.2.2 BRAF

BRAF是KRAS下游的丝氨酸/苏氨酸激酶，将RAS鸟苷三磷酸连接到丝裂原活化蛋白激酶家族的下游蛋白，控制细胞增殖。RAF激酶家族包括3个成员：A-RAF、B-RAF和RAF-1（也称为C-RAF）^[13]^。体细胞*BRAF*突变最初是在黑色素瘤中被发现，约80%的突变影响激酶结构域中外显子15的Val600残基。NSCLC中*BRAF*突变占1%-3%，绝大多数是在腺癌^[14]^。Paik等^[15]^检测697例肺腺癌患者*BRAF*的突变率为3%，有突变的患者都是吸烟或既往吸烟者，V600E是最常见的突变，占50%，位于外显子15，其它突变类型有G469A（39%）、D594G（11%），分别位于外显子11和外显子15。*BRAF*突变与酶活性增加相关，导致MAPK2和MAPK3组成型活化。现已研发出多个BRAF抑制剂（PLX4032、XL281、selumetanib、GSK2118436），其中最有前景的复合物是PLX4032（vemurafenib），它是选择性BRAF小分子抑制剂，对BRAF V600E突变阳性的黑色素瘤有非常明显的疗效^[16]^，其对肺癌的疗效还有待评价。

#### 1.2.3 丝裂原活化蛋白激酶（motigen-activated protein kinase, MAP2K1）

也称为MEK1，是一种丝氨酸/苏氨酸激酶，位于BRAF的下游，活化B-RAF下游的MAPK2、MAPK3。现已明确肿瘤中MAP2K1有3种突变：*Q56P*、*L57N*和*D67N*，所有突变均发生在蛋白的非激酶部分。NSCLC中*MAP2K1*突变率为1%，主要是在腺癌。

#### 1.2.4 PTEN（phosphatase and tensin homologue）

*PTEN*是抑癌基因，通过将PIP3去磷酸化抑制AKT活化来拮抗PI3K/AKT信号通路。*PTEN*缺失突变或未活化导致*PIK3CA*基因自身功能增强，PI3K/AKT发生组成型活化，激活*RAS*癌基因。PTEN缺失在鳞癌中多于腺癌。此外，PTEN缺失伴随磷酸化AKT过表达，与预后差相关^[17]^。近来研究^[18]^证实PTEN可保护基因组的稳定性。也有研究^[19]^报告PTEN与TKI耐药有关。现有临床试验评估PI3K抑制剂在PTEN缺失肿瘤中的疗效。

### 1.3 PI3K/AKT/mTOR通路

#### 1.3.1 PIK3CA

是脂类激酶，再合成磷脂酰肌醇-3-磷酸盐，后者是生长受体和细胞内下游信号通路的关键介质。PI3K以异二聚体形式存在，包括1个催化亚单位和1个调节亚单位，是细胞增殖和凋亡的重要调节因子。PI3K由三类复杂的家族成员组成，每一类有多个亚单位和亚型，PI3Kα是这个家族中研究最多的一种酶。PIK3CA编码Ⅰ类PI3K的p110α催化亚单位。30%恶性胶质瘤、胃癌中可见*PIK3CA*突变，但在肺癌中极少约2%^[20]^。30%鳞癌和6%腺癌中可见到基因扩增^[21]^。PIK3CA最常见的两个突变区域是外显子9和20，分别编码蛋白螺旋和激酶结构域。这些突变导致液态激酶活性增强及PI3K/AKT信号通路的组成型活化。NSCLC中*PIK3CA*突变最常影响外显子9的编码催化结构域的E542和E545残基，这些突变在鳞癌中较腺癌多见，常发生在*EGFR*突变的肿瘤^[22]^。体外实验显示*PIK3CA*突变的肿瘤对EGFR耐药。小分子抑制剂BEZ235以PI3K、mTOR蛋白为靶点，在小鼠中已显示出抗肿瘤活性。多个PI3K抑制剂已进入早期临床试验，疗效有待进一步被验证。

#### 1.3.2 AKT

又名蛋白激酶B，是PI3K下游的丝氨酸/苏氨酸激酶，被PI3Kα激活，调节PI3K信号通路。*AKT1*基因中一种主要的频发突变为E17K，已在乳腺癌、结肠癌、卵巢癌等几种实体瘤中被鉴定^[23]^，NSCLC中突变率为1%^[24]^，仅在鳞癌中被鉴定，与抗凋亡有关。对EGFR-TKI有效的病例中可见AKT磷酸化增强，AKT可能被*EGFR*突变活化。蛋白激酶B抑制剂MK2206已进入Ⅰ期临床试验。

#### 1.3.3 mTOR

是一种丝氨酸/苏氨酸激酶，作为雷帕霉素的细胞内靶点被鉴定。mTOR通过AKT磷酸化而激活，促进细胞增殖。mTOR的上游调节因子是PI3K/Akt通路，激活mTOR对生长因子发生反应，通过真核启动因子4E结合蛋白1和40S核糖体蛋白S6激酶两种不同通路参与对翻译的调节^[25]^，在EGFR信号传导通路中起抗凋亡的作用。现已研发出几种抑制剂，并取得了一定的疗效。

### 1.4 人类表皮生长因子受体2（human epidermal growth factor receptor 2, HER2）

*HER2*突变在NSCLC中约为2%，为外显子20插入突变，最常见于776密码子Tyr-Val-Met-Aal序列，与HER2激酶组成型活化有关，导致下游效应器如AKT、MEK磷酸化。*HER2*突变在不吸烟、女性、亚裔患者中多见，腺癌明显多于其它NSCLC^[26]^。*HER2*突变与*EGFR*或*KRAS*突变互斥。曲妥珠单抗在治疗HER2阳性乳腺癌取得了非常明显的疗效，但早期临床试验中它对非选择NSCLC中仅表现出微乎其微的作用。基础研究^[27]^显示，携带突变基因的细胞对作用于EGFR和HER2的药物，如拉帕替尼，一种小分子酪氨酸激酶抑制剂治疗敏感，但对只作用于EGFR的抑制剂不敏感。表达HER2突变的转基因鼠发生肺腺癌，在BIBW 2992（作用于EGFR和HER2的抑制剂）与雷帕霉素（mTOR抑制剂）联合作用下，肿瘤明显缩小。与此类似，另一项试验^[28]^中HER2突变阳性的肺腺癌患者接受BIBW 2992治疗也获得了较好的疗效。

### 1.5 LKB1

LKB1以前也称之为丝/苏氨酸蛋白激酶11（serine theronine protein kinase 11, STK11）。1997年*LKB1*基因突变首次被报告，位于染色体19p13.3，可引起peutz-jeghers综合征（黑色素斑-胃肠多发性息肉综合征）^[29]^。患有此综合征患者易患各器官的多种肿瘤，其中胃肠道肿瘤最常见。*LKB1*突变在NSCLC中多见，20%-30% NSCLC有*LKB1*突变，腺癌中突变率最高（白种人约30%、韩国人8%、日本人7%）^[30]^。*LKB1*基因被认为是一个抑癌基因，与p53和CDC42相互作用，调节AMP活化蛋白激酶的活性。LKB1其它抑制肿瘤的特性可能包括抑制mTOR介导、调节细胞极性和抑制细胞周期及p53活性。2008年Koivunen等^[31]^对310例NSCLC肿瘤标本的检测发现*LKB1*突变多见于腺癌（13%），明显多于鳞癌（5%），*LKB1*突变与吸烟、*KRAS*突变相关，与EGFR互斥。

### 1.6 MET（mesenchymal-epithelial transition factor）

MET编码肝细胞生长因子受体（hepatocyte growth factor receptor, HGFR），是一种酪氨酸激酶膜受体，位于染色体7q21-q31。MET受体或肝细胞生长因子（hepatocyte growth fator, HGF）及它的配体HGF可触发胞内关键的信号级联，如RAS/RAF/MEK、PI3K/AKT/mTOR、Rho、Rac1、CDC42等^[32]^。通过HGF或MET过表达、*MET*基因扩增、突变，发生HGF/MET调节异常，导致细胞增殖、侵袭、迁移及血管生成。与肾癌和胃癌不同，MET激酶结构域突变在NSCLC较为罕见。Ding等的研究^[33]^发现，188例肺腺癌中，仅确定3例有*MET*突变，2个发生在编码近膜域的外显子13上，1个在编码激酶结构域的外显子18上，这些突变的意义目前尚是未知的。队列研究发现既往未接受过EGFR-TKI治疗的NSCLC患者中MET扩增约为1.4%-21.0%，数据范围较大的原因与不同研究采用的方法和截断值有关。MET扩增可见于鳞癌和腺癌。MET扩增与EGFR-TKI继发耐药有关^[34]^，有报道约20%TKI获得性耐药患者肿瘤中有*MET*基因扩增，驱动HER3依赖的PI3K活化。研究^[35]^报告HGF可能促进*MET*基因的扩增，与新的TKI耐药机制有关。现已有开发的HGFR小分子抑制剂。

### 1.7 *EML4*-*ALK*融合基因

2007年日本学者首先报告在NSCLC患者中发现*EML4*-*ALK*融合基因^[36]^。*ALK*基因编码的酪氨酸受体是一跨膜蛋白，胰岛素受体酪氨酸激酶家族的成员，其生理功能尚不清楚。正常情况下仅在中枢神经系统、小肠、睾丸等组织中有表达，正常肺组织不表达。EML4也位于2号染色体，其易位形成*EML4*-*ALK*融合基因，导致酪氨酸激酶结构域组成型二聚体化及非配体依赖性活化，激活下游的MAPK1和PIK3CA/AKT1信号通路，致细胞增殖、侵袭、凋亡抑制，ALK通路的下游效应器包括RAS活化蛋白、MAPK1、PIK3CA和STAT3信号通路^[37]^。研究发现EML4的外显子1-13与ALK的外显子20-29相融合。迄今已发现了15种EML4-ALK不同的基因变异体，这些变异体均有功能，最多见的是变异体1和变异体3a，分别在33%和29%患者中检测到^[38]^。

在非选择NSCLC患者中，EML4-ALK易位的频率在3%-7%，人种间无明显差异。几项研究显示，临床特征中年龄与ALK重排相关性最强，携带ALK重排患者的中位年龄明显低于阴性患者，但不同性别间ALK频率无差异，吸烟是否与其相关也不一致。*EML4*-*ALK*融合基因的频率在肺腺癌中多见，腺泡、筛子形、印戒细胞等组织学类型中多见^[39]^。EML4-ALK常在EGFR和KRAS野生型患者中表达，与EGFR、KRAS互斥。Shaw等^[40]^在141例NSCLC队列中，采用FISH方法发现不吸烟/轻度吸烟/EGFR阴性患者中ALK阳性率达33%。台湾Wu等^[41]^发表的研究中通过对有胸腔积液的肺腺癌患者的胸水检测*EGFR*突变，*EGFR*基因野生型患者通过RT-PCR检测ALK的阳性率为34%。多项研究报告的阳性率相差较多，与研究样本量的大小、样本是否经过筛选、检测方法等因素有关。*EML4*-*ALK*融合基因的检测有3种方法，即FISH、IHC和逆转录-聚合酶链反应（reverse transcription-polymerase chain reaction, RT-PCR），NCCN推荐FISH用于检测ALK，其敏感性和特异性均较好，是Ⅰ期、Ⅱ期、Ⅲ期克唑替尼临床试验中采用的检测方法，是目前检查融合基因的金标准。免疫组化的敏感性低，尚需进一步的标准化，RT-PCR虽然敏感性高，但需在新鲜组织中检测。

克唑替尼是ALK和MET抑制剂。Ⅰ期试验中82例（71%为复治）ALK阳性NSCLC患者接受克唑替尼，有效率为61%，疾病控制率88%，中位PFS为10个月，中位生存期还未达到^[42]^，克唑替尼在很短时间内被美国FDA批准上市，用于ALK阳性的晚期NSCLC治疗。Ⅱ期研究（PROFILE 1005）中，ALK阳性NSCLC患者接受克唑替尼治疗，有效率为50%。Ⅲ期研究（PROFILE 1014, PROFILE 1007）正在进行中。

### 1.8 Eph（erythropoietin producing human hepatocellula carcinoma cell line）

基础研究显示Eph是受体酪氨酸激酶（receptor tyrosine kinase, RTKs）家族最大的一个亚群，有促进肿瘤生长、侵袭、转移和肿瘤新生血管形成的作用。Eph受体包括15个受体和9个配体，分为两类，即A类和B类。A类中的EphA2和EphA3与肺癌关系较为密切。NSCLC肿瘤EphA2高表达与吸烟史、转移相关，尤其是脑转移，高表达患者预后差，EphA2被视为一个独立的肺癌预后标志。肺癌细胞中*EphA3*突变最常见，可能与肿瘤的驱动有关。肺癌患者中也发现B类Eph，EphB3过表达与NSCLC肿瘤大小、分化、转移有关。现已开发出以Eph为靶点的抗肿瘤药物^[43]^。

## 2 几项驱动基因筛查研究

Ding等^[33]^于2008年发表了第一个大样本的肿瘤基因筛查研究，在188例原发肺腺癌中筛查了623个候选基因，该研究除了筛查出肺腺癌已知的频率较高的几种突变，包括抑癌基因*TP53*、*CDKN2A*、*STK11*，癌基因*KRAS*、*EGFR*，以及几种频率较低的基因*PTEN*、*NRAS*、*ERBB2*、*BRAF*、*PIK3CA*，此外，还发现几种新的突变基因：抑癌基因*NF1*、*RB1*、*ATM*、*APC*，原癌基因*ERBB4*、*KDR*、*FGFR4*和*NTRK*。这项研究还对不同吸烟状况、肿瘤分化程度、肿瘤分期、组织学亚型等临床特征下突变的分布做了分析。

Kris等^[44]^在2011年ASCO上发表了来自LCMC（NCI’s Lung Cancer Mutation Consortium）的一项研究，对1, 000例肺腺癌标本中10种驱动基因进行检测。全部患者为Ⅲb期/Ⅳ期，PS评分为0分-2分，有足够的组织标本。研究共入组830例患者，60%患者有驱动基因突变。各基因频率分别为：KRAS 25%、EGFR 23%，ALK重排6%、BRAF 3%、PIK3CA 3%、MET扩增2%、HER2 1%、MEK1 0.4%、NRAS 0.2%、AKT1 0，约36.4%肺腺癌不携带已知突变。95%的基因突变是互斥的。目前采用多重分析检测肺癌全部突变已成为半数LCMC机构病理科的常规。

美国马萨诸塞州总医院癌症中心的Sequist等^[45]^于2011年发表了1篇论文，采用多重PCR及SNaPshot测序对552例NSCLC患者福尔马林固定石蜡包埋的组织标本进行了多基因的检测，另外采用FISH检测*ALK*融合基因。全部患者中51%有驱动突变，常见的为*KRAS*、*EGFR*、*ALK*。各基因的频率分别为：*KRAS* 24%、*EGFR* 13%、*ALK* 5%、*PIK3CA* 4%。*PIK3CA*在鳞癌中多见，*KRAS*突变患者较野生型患者生存差（16.4个月 *vs* 22.5个月），*EGFR*突变患者生存优于野生型（34.3个月 *vs* 20.0个月）。研究显示全面的基因分型更有效地符合NSCLC的临床实践，在影响治疗决策和指导患者参与相关临床试验上有很大的实用性。

我国Xu等^[46]^采用SurPlex^®^-xTAG70plex平台对随机选择的861例中国NSCLC患者进行*EGFR*、*KRAS*、*BRAF*、*PIK3CA* 4个基因的检测，4个基因的频率分别为41.0%、8.0%、0.7%和3.7%。*EGFR*外显子19、20、21突变在女性中高于男性、腺癌高于其它病理类型、不吸烟者高于吸烟者。*KRAS*突变在男性中多于女性，腺癌多于其它类型，吸烟者多于不吸烟者；PIK3CA在腺癌中低于其它肺癌类型；全部患者中仅1例患者同时携带*EGFR*和*KRAS*突变。

2012年ASCO上，Yang等^[47]^在2012年ASCO上报告了PIONEER研究结果，即亚洲晚期肺腺癌*EGFR*突变的分子流行病学调查。研究采用Scorpion ARMS（TheraScreen^®^ EGFR29 Mutation Test Kit）对新确诊的Ⅲb期/Ⅳ期肺腺癌组织标本进行*EGFR*突变检测。共入组来自7个国家和地区的1, 482例患者，肺腺癌*EGFR*基因的总突变率为51.4%，各国或地区的*EGFR*突变率分别为：越南64.2%（77/120）、台湾62.1%（108/174）、泰国53.8%（63/117）、菲律宾52.3%（34/65）、中国50.2%（372/741）、香港47.2%（76/161）、印度22.2%（16/72）；女性突变率61.1%，男性44.0%；不吸烟者突变率为60.7%，重度吸烟31.4%。人种（*P*=0.001）和吸烟包/年（*P*=0.001）是影响突变最重要的因素，吸烟状况调整后的不同年龄间突变率无差异。

上述几项研究中各队列的驱动基因的频率并不相同，这与队列的选择直接相关，采用的检测方法不同也会对结果有轻微的影响，不同的人种间的有些基因频率相差较大，如*EGFR*、*KRAS*在东西方患者差异较大，同一人种的基因频率也可能因性别、吸烟状况、年龄等而有不同。

## 3 展望

目前的靶向治疗虽得到一定的疗效，但总体上疗效并不是非常理想，因为肿瘤的形成是一个多基因参与的异常复杂的过程，现有的靶向药物如小分子激酶抑制剂多是针对一个靶点而设计，现也仅发现约50%肺腺癌患者携带不同的驱动基因，还有相当多的驱动基因未被发现，因此只有发现更多的驱动基因，阐明基因之间的相互作用关系，开发出更多的靶向药物，进一步明晰药物的代谢作用，联合应用或联合其它的治疗方式，才有可能收到理想的疗效。而且，大多数患者接受靶向药物治疗后会出现耐药问题，耐药机制尚未十分明确，耐药后的治疗方案也不十分理想。单一或少数几个基因检测已不能满足分子靶向治疗的要求，因此开发敏感性和特异性均较好的、高通量的能够同时检测融合、点突变、插入、缺失和扩增等多种基因异常的新平台，将更能适应临床治疗策略的需要。此外，临床上许多患者是通过细胞学或微小的活检诊断，标本不能满足多基因的检测，因此，在临床实践中需加强多学科间合作，不断提高肿瘤科、胸外科和呼吸科医生等对组织学标本重要性的认识。总之，真正实现肺癌的个体化治疗仍然任重而道远。
